# A glistening pink papule on the shoulder of a patient with HIV: poroid hidradenoma

**DOI:** 10.1093/skinhd/vzag058

**Published:** 2026-04-30

**Authors:** Mohammed Shanshal, Muna Abuayyash

**Affiliations:** Department of Dermatology, Imperial College London, Imperial College Healthcare NHS Trust, London, UK; Department of Histopathology, Imperial College Healthcare NHS Trust, London, UK

## Abstract

A 69-year-old man living with HIV presented with a tiny, glistening pink papule on the shoulder with nonspecific dermoscopy, but histology revealed a poroid hidradenoma, the rarest poroid neoplasm. This image highlights the subtle clinical and dermoscopic clues to this adnexal tumour and underscores the need for histological assessment and complete excision of changing papules in patients who are immunocompromised.

Dear Editor, A 69-year-old man with HIV receiving antiretroviral therapy (emtricitabine/tenofovir alafenamide plus raltegravir), with HIV-1 viral load <20 copies mL^–1^ and most recent available CD4^+^ lymphocyte count of 507 cells µL^–1^, presented with a 2-year history of a papule on his posterior shoulder, which had recently enlarged and become pruritic and tender. Clinical examination revealed a well-circumscribed, glistening, dome-­shaped pink papule measuring approximately 2–3 mm. Dermoscopy demonstrated a symmetric, structureless, pink–red ovoid area with subtle bluish translucency and a small inferior cluster of dotted–glomerular vessels (Figure 1a, b).

**Figure 1:**
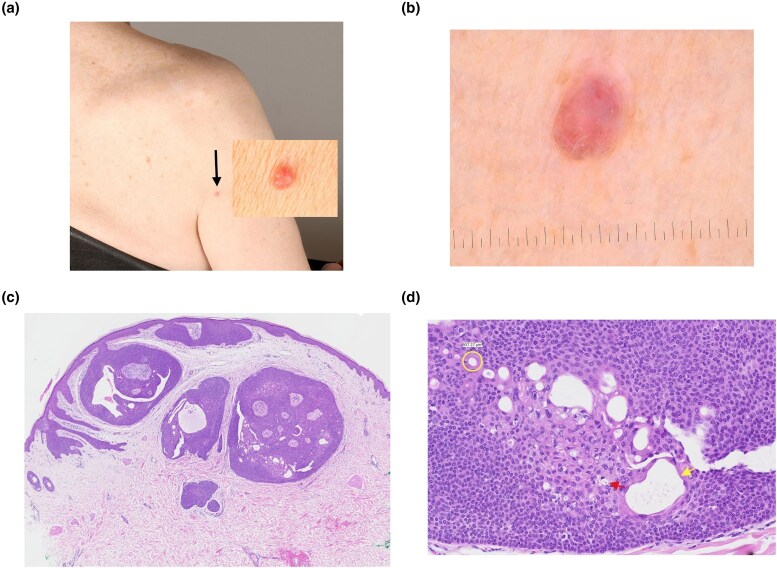
(a) Clinical photograph of the posterior right shoulder showing a tiny dome-shaped pink papule (black arrow; inset, close-up view). (b) Dermoscopy reveals a symmetric, structureless pink–red ovoid lesion with subtle bluish translucency and a small inferior cluster of dotted–glomerular vessels (scale in mm). (c) Haematoxylin and eosin, original magnification ×20: low-power view showing a well-circumscribed, multilobulated intradermal epithelial tumour composed of solid and cystic nodules. (d) Haematoxylin and eosin, original magnification ×200: higher-power view of the solid and cystic components, demonstrating poroid cells admixed with glycogen-rich clear cells with eccentric nuclei (red arrow), a small ductal lumen bordered by a dense eosinophilic cuticle (circle) and a cystically dilated sweat-duct space containing eosinophilic secretions (yellow arrow), in keeping with poroid hidradenoma.

Histopathological examination following complete excision revealed a well-demarcated, multilobulated intradermal ­epithelial neoplasm. The tumour exhibited a dual architecture, ­composed of solid nodules and cystic spaces. The solid component consisted of monomorphic poroid cells admixed with glycogen-rich clear cells containing eccentric nuclei. The cystic component featured ductal lumina bordered by a dense eosinophilic cuticle and cystically dilated sweat-duct spaces containing eosinophilic secretions. These findings were diagnostic of poroid hidradenoma (Figure 1c, d).

Poroid hidradenoma is the rarest variant of poroid neoplasms, defined by the solid-cystic architecture of a hidradenoma combined with the distinct cytology of an eccrine poroma.^1,2^ While typically benign, these adnexal tumours carry a very low (<1%) risk of malignant transformation.^1,3^ In patients who are immuno­compromised, the clinical differential for such a lesion is broad, but the bland dermoscopic features can be misleading. Simple local excision is the standard management, providing a definitive diagnosis and curative treatment, with rare recurrence if completely excised.^1–4^ This case underscores the principle that even a small, clinically unremarkable papule in a patient who is immunocompromised host warrants a high index of suspicion and necessitates histological confirmation to exclude underlying neoplasia.

## Data Availability

Not applicable.

## References

[1] Lim JS, Kwon ES, Myung KB, Cheong SH. Poroid hidradenoma: a two-case report and literature review. Ann Dermatol 2021; 33:289. 34079192 10.5021/ad.2021.33.3.289 PMC8137338

[2] Brooks A, Morris M, Cuda J et al Poroid hidradenoma: case report and comprehensive review of the literature. Case Rep Dermatol 2023; 15:202–16. 37928337 10.1159/000531052 PMC10620550

[3] Efared B, Boubacar I, Ousmane Kadre KA et al Poroid neoplasms: a clinicopathological study of 13 cases. Clin Pathol 2024; 17:2632010X241281460. 10.1177/2632010X241281460 PMC11402061 39282157

[4] Lupu M, Tebeica T, Malciu AM, Voiculescu VM. Poroid hidradenoma: dermoscopic and in vivo reflectance confocal microscopic description. Diagnostics 2022; 12:255. 35204346 10.3390/diagnostics12020255 PMC8871167

